# Histopathological Evaluation of Deceased Persons in Lusaka, Zambia With or Without Coronavirus Disease 2019 (COVID-19) Infection: Results Obtained From Minimally Invasive Tissue Sampling

**DOI:** 10.1093/cid/ciab858

**Published:** 2021-12-15

**Authors:** Victor Mudenda, Chibamba Mumba, Rachel C Pieciak, Lawrence Mwananyanda, Charles Chimoga, Benard Ngoma, Zacharia Mupila, Geoffrey Kwenda, Leah Forman, Rotem Lapidot, William B MacLeod, Donald M Thea, Christopher J Gill

**Affiliations:** 1 University Teaching Hospital, Department of Pathology, Lusaka, Zambia; 2 Boston University School of Public Health, Department of Global Health, Boston, Massachusetts, USA; 3 Right to Care Zambia, Lusaka, Zambia; 4 University of Zambia, School of Health Sciences, Department of Biomedical Sciences, Lusaka, Zambia; 5 Boston University School of Public Health, Biostatistics and Epidemiology Data Analytics Center (BEDAC), Boston, Massachusetts, USA; 6 Boston University School of Medicine, Boston, Massachusetts, USA

**Keywords:** autopsy, COVID-19, minimally invasive tissue sampling, pathology, postmortem

## Abstract

**Background:**

Although much has been learned about the pathophysiology of coronavirus disease 2019 (COVID-19) infections, pathology data from patients who have died of COVID-19 in low- and middle-income country settings remain sparse. We integrated minimally invasive tissue sampling (MITS) into an ongoing postmortem surveillance study of COVID-19 in deceased individuals of all ages in Lusaka, Zambia.

**Methods:**

We enrolled deceased subjects from the University Teaching Hospital Morgue in Lusaka, Zambia within 48 hours of death. We collected clinical and demographic information, a nasopharyngeal swab, and core tissue biopsies from the lung, liver, and kidneys for pathologic analysis. Individuals were considered eligible for MITS if they had a respiratory syndrome prior to death or a COVID-19+ polymerase chain reaction (PCR) nasopharyngeal swab specimen. Samples were retested using quantitative reverse transcriptase PCR.

**Results:**

From June to September 2020 we performed MITS on 29 deceased individuals. PCR results were available for 28/29 (96.5%) cases. Three had a COVID-19+ diagnosis antemortem, and 5 more were identified postmortem using the recommended cycle threshold cut-point <40. When expanding the PCR threshold to 40 ≤ cycle threshold (Ct) ≤ 45, we identified 1 additional case. Most cases were male and occurred in the community The median age at death was 47 years (range 40–64). Human immunodeficiency virus (HIV)/AIDS, tuberculosis, and diabetes were more common among the COVID-19+ cases. Diffuse alveolar damage and interstitial pneumonitis were common among COVID-19+ cases; nonspecific findings of hepatic steatosis and acute kidney injury were also prevalent in the COVID-19+ group. Vascular thrombi were rarely detected.

**Conclusions:**

Lung abnormalities typical of viral pneumonias were common among deceased COVID-19+ individuals, as were nonspecific findings in the liver and kidneys. Pulmonary vascular thrombi were rarely detected, which could be a limitation of the MITS technique. Nonetheless, MITS offers a valuable alternative to open autopsy for understanding pathological changes due to COVID-19.

Since the first reported cases of the 2019 novel severe acute respiratory syndrome coronavirus 2 (SARS-CoV-2) in Wuhan, China, coronavirus disease-19 (COVID-19) has spread globally with devastating consequences [1, 2]. The World Health Organization currently estimates that there have been ~150 million COVID-19 cases and ~3 million COVID-19-related deaths globally [3]. The first cases of COVID-19 in Zambia were detected in March 2020 [4]. The COVID-19 surveillance and testing efforts in Zambia remain ongoing [5]. Recently, our team reported that between 15% and 19% of deceased individuals tested positive for COVID-19 when tested postmortem, and the majority of these individuals had typical symptoms of COVID-19 prior to death [6].

The clinical presentation of patients with COVID-19 varies widely, with syndromes affecting respiratory, cardiac, neurological, renal, and hepatic systems. In most cases acute respiratory distress appears to be the main debilitating disorder, but there have been reports of metabolic derangement, thrombotic events and shock as complications [7, 8]. Diagnostic autopsies provide an understanding of the pathological and pathophysiological mechanisms of COVID-19, and yet very few studies from African sites have reported on these findings in laboratory-confirmed COVID-19 deaths [9]. Over the last several years, clinician and family attitudes toward autopsies, lack of resources, and cost has led to a decline in autopsy rates. At the onset of the COVID-19 pandemic, these barriers were further compounded by the fear of contracting COVID-19 while conducting an autopsy, resulting in a scarcity of valuable, postmortem data [10].

An alternative to standard open autopsy is minimally invasive tissue sampling (MITS), a technique that uses core biopsy needles to sample different organs [11]. A theoretical advantage to MITS is that it may be more acceptable to family members than open autopsies [12]. Emerging data support the accuracy and validity of MITS for investigations of infectious etiologies [13–15]. An early report of 4 patients in South Africa suggests that MITS may also be valuable for studying the pathology of COVID-19 infections [16].

In order to link COVID-19 polymerase chain reaction (PCR) status with pathologic evidence for COVID-19 disease, we integrated MITS into an ongoing, postmortem COVID-19 surveillance study. As previously reported by our team, COVID-19 occurred in nearly 20% of deceased individuals in our cohort and occurred across the age spectrum [6].

In this substudy, we collected lung, liver, and kidney biopsies on a subset of participants who were clinically suspected to have COVID-19 based on presenting respiratory symptoms or who were known to be COVID-19 infected based on antemortem testing. The current analysis describes the observed pathology in this subset. These results are contrasted against a group of individuals who had also died of a respiratory syndrome but were determined to be COVID-19- by PCR.

## METHODS

### Enrollment of Cases

From August 2017 to August 2020, our team enrolled deceased infants aged 4 days to 6 months into the Zambian Pertussis RSV Infant Mortality Estimation (ZPRIME) study in order to measure the fatal impact of respiratory syncytial virus (RSV) and *Bordetella pertuss*is in young infants. In June 2020, and in direct response to the COVID-19 pandemic, our team amended our ZPRIME study protocol to enroll deceased people of all ages and to test for SARS-CoV-2 infection by PCR.

All cases were enrolled from the University Teaching Hospital (UTH) in Lusaka, Zambia, within 48 hours of death. Consent was obtained from the deceased’s next of kin when they came to the morgue to claim the body. Participants who died while receiving care from UTH were classified as “facility deaths.” All other deaths were classified as “community deaths.” For each participant we collected demographic and clinical data and a nasopharyngeal swab. Antemortem COVID-19 test results were recorded (when applicable).

After these data were recorded, a ZPRIME study team member assessed if the deceased person met the eligibility criteria for the MITS substudy. The deceased was considered eligible for MITS if she/he experienced cough, fever, shortness of breath, and/or difficulty breathing prior to death or if there was a positive COVID-19 test antemortem. After obtaining informed consent, a trained ZPRIME study member conducted MITS, recording the date and time of the procedure and samples collected.

### Sample Collection, Testing, and Storage

#### Nasopharyngeal Samples

Nasopharyngeal samples were collected from each nares using flocked tipped nylon swabs. Immediately after collection, all swabs were placed into 3-mL universal transport media and stored at 4–8°C until they could be transported to our on-site PCR testing lab. All samples received at the lab were aliquoted and stored at −80°C.

Total nucleic acid was extracted from the samples using the NucliSense EASYMAG system [17, 18]. We used the kit developed by the US Centers for Disease Control to perform reverse transcriptase quantitative PCR to identify SARS-CoV-2. We included positive and negative controls and the constitutive human enzyme, RNase P, on each assay plates to validate the adequacy of sample collection and the absence of PCR inhibitors.

#### Tissue Biopsies

Following the next of kin’s consent to the MITS substudy, a ZPRIME staff member scanned the unique MITS kit subject ID into the REDCap data collection system. This subject ID was then linked to the ZPRIME enrollment ID so that the demographic, clinical, and nasopharyngeal sample data could be electronically linked across the 2 studies.

The body and MITS kit were then transported to the autopsy theatre within the UTH Morgue where the team could collect the tissue samples.

Team members wore N95 masks, gowns, single use booties, and surgical gloves during all sampling procedures. After disinfecting the surface of the body with ethanol and iodine, core tissue biopsies from the lungs, liver, and kidneys were collected using 16-gauge biopsy needles. Two biopsies from the upper, middle, and lower left and right lungs were collected, for a total of 12 lung samples for each case. Two biopsies were attempted each from the liver and right and left kidneys. Half of the samples were put into universal transport media and sent to our molecular lab for frozen storage and later PCR testing (data not presented in this article). The other half of the samples were fixed in 10% buffered formalin and transported to our pathology lab. Tissues were kept in formalin for a minimum of 24 hours before routine processing and paraffin embedding. The paraffin blocks were then cut and stained with hematoxylin and eosin.

Interpretation of the histologic samples was conducted by 2 pathologists (C.M. and V.M.) working independently and blinded to the PCR results. Discrepancies were resolved by internal discussion yielding a final interpretation of each blinded set of participant specimens.

## RESULTS

Between June and September 2020 we collected core tissue samples from 29 deceased persons using MITS. PCR results were available for 28/29 (96.5%) subjects. SARS-CoV-2 was detected in 9/28 (32%) individuals, leaving 19 (68%), where COVID-19 was neither identified antemortem nor postmortem. Three cases had a positive COVID-19 diagnosis antemortem, and 5 more were identified by postmortem PCR testing using kit manufacturer’s recommended cycle threshold (Ct) cut-point of <40. When expanding the PCR threshold to any detection (ie, 40 ≤ Ct ≤ 45), we identified one additional case. We elected to include all 9 of these cases under the assumption that a low intensity PCR signal was more likely a true positive in a patient with a low or declining viral load than a false positive.

The majority of deaths were community deaths (55%, 16/28) and male (68%, 19/28). The median age at death was 47 years old (range 40–64), but cases who were COVID-19+ tended to be younger (Table 1).

**Table 1.** Demographic and Clinical Characteristics of MITS Cases

Parameter	COVID-19−	COVID-19+	All
	N = 19	N = 9	N = 28
Females, no. (%)	6 (32%)	3 (33%)	9 (32%)
Median age at death, years (IQR)	54 (36–65)	46 (42–61)	47 (40–64)
Community Deaths^a^, no. (%)	12 (63%)	4 (44%)	16 (55%)

Abbreviations: COVID-19, coronavirus disease 2019; IQR, interquartile range; MITS, minimally invasive tissue sampling.

a Community deaths were those that occurred outside of a medical facility.

Table 2 summarizes the symptoms that the deceased experienced leading up to her/his death and underlying risk factors for severe disease. These were collected from the deceased’s next of kin and/or the medical chart (for facility deaths only).

**Table 2.** Demographic Features, Presenting Symptoms, and Underlying Conditions of COVID-19 Positive and Negative Participants

Case No.	Location( Community v. Facility)	Age at Death, Years	Sex	Cough	Difficulty Breathing	Fever	Shortness of Breath	Other^a^	Underlying conditions present ^┼^
COVID-19+									
1	Facility	79	Female	X	X	X	X		HIV/AIDS
2	Facility	38	Female	X		X			HIV/AIDS, TB
3	Facility	65	Male	X	X	X	X		Alcohol misuse, DM, COPD, smoking
4	Facility	48	Male	X	X	X	X		Cancer, HIV/AIDS, TB
5	Facility	42	Male	X	X		X		Alcohol misuse, CVD, smoking, TB
6	Community	61	Male	X		X		X	HIV/AIDS, Malnutrition, TB
7	Community	40	Male	X	X		X	X	DM, HIV/AIDS, HTN
8	Community	45	Male	X	X			X	DM, HIV/AIDS, HTN, TB
9	Community	46	Female	X	X	X		X	HTN, TB
COVID-19−									
10	Facility	67	Male	X	X				HIV/AIDS, HTN, Malaria
11	Facility	55	Male	X	X	X			COPD, CVD, HIV/AIDS
12	Facility	56	Male	X	X	X			Alcohol misuse, HIV/AIDS, HTN, smoking, TB
13	Facility	21	Male	X	X		X		COPD, HIV/AIDS, malnutrition, smoking, TB
14	Community	72	Male	X	X		X		COPD, smoking, TB
15	Community	40	Female		X				HTN
16	Facility	83	Female	X	X				HTN, smoking
17	Facility	66	Male	X	X				Alcohol misuse, COPD, malnutrition, TB,
18	Community	0.13	Female	X	X	X			
19	Community	54	Male	X	X	X			HIV/AIDS, TB
20	Community	18	Male	X	X	X	X		
21	Community	64	Female		X		X		
22	Community	40	Male	X	X	X	X		Alcohol misuse, COPD, TB
23	Community	73	Male	X	X		X		Alcohol misuse, smoking, TB
24	Community	58	Female	X	X				TB
25	Community	33	Male		X	X			DM
26	Facility	38	Male		X	X	X		Alcohol misuse, HIV/AIDS
27	Community	40	Male	X	X		X		Smoking, TB
28	Community	33	Female	X	X	X			Alcohol misuse, TB

Abbreviations: COPD, chronic obstructive pulmonary disease; COVID-19, coronavirus disease 2019; CVD, cardiovascular disease; DM, diabetes mellitus; HIV/AIDS, human immunodeficiency virus/acquired immunodeficiency syndrome; HTN, hypertension; TB, tuberculosis.

a Includes fast breathing, headaches, achy joints/muscles, chest pain, vomiting, diarrhea, runny nose, nausea, sore throat, loss of taste/smell, hemiparesis, and sudden onset abdominal pain.

Because MITS eligibility was based on the presence of cough, fever, shortness of breath, and/or difficulty breathing, all participants had 1 or more of these symptoms. “Difficulty breathing” and “Cough” were reported in the majority of cases, 93% and 86%, respectively. All 9 (100%) of the MITS cases that were COVID-19+ reported “cough” as a symptom preceding death. By comparison, among the 19 COVID-19− individuals, only 74% (14/19) had cough, 53% (10/19) had fever, and 42% (8/19) had shortness of breath. These proportions were similar to the COVID-19+ group, and none of these differences achieved statistical significance. Other symptoms such as fast breathing, headaches, myalgia/arthralgia, chest pain, vomiting, diarrhea, nausea, hemiparesis, anosmia, and sudden abdominal pain had been reported in 5/9 (56%) COVID-19+ cases and in none of the COVID-19− cases (0/19; 0%). What clearly distinguished the 2 groups were the symptoms identified among COVID-19+ cases; these were more specifically associated with the COVID-19 syndrome, notably anosmia, gastrointestinal (GI) pain, and symptoms suggestive of acute cardiovascular and neurologic infarcts.

The most common comorbid conditions occurring in at least 25% of the MITS cases, regardless of COVID-19 status were tuberculosis (57%), human immunodeficiency virus (HIV)/AIDS (43%), smoking (36%), alcohol misuse (29%), and hypertension (25%). Although our sample size is small, several conditions that appeared to be more common in the COVID-19+ group were HIV/AIDS (PR 2.11, 95% confidence interval [CI] .94–4.73); tuberculosis (PR 1.27, 95% CI .68–2.38); and diabetes (PR 6.3, 95% CI 0.76-52.3).

For each MITS participant we successfully collected lung, liver, and kidney tissue biopsies from 100% (28/28), 96% (27/28), and 71% (20/28) of participants, respectively. We had samples from all 3 organs in 20/28 cases. Of the participants with complete sample sets, 6/20 cases had evidence of abnormal pathology across all 3 tissue samples; 3 of these cases were COVID-19+ cases. The remaining 14 cases for which we had complete MITS sets showed variable and nondiagnostic pathology. Histological findings from the lung, liver, and kidney are presented below (Supplementary Table 1).

### Lung

**Figure 1.**
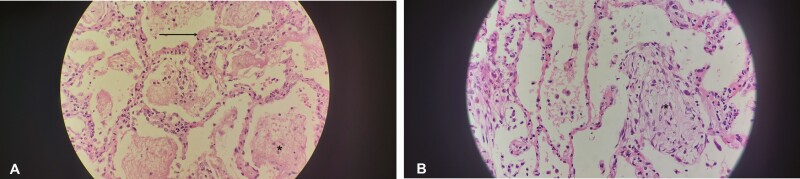
Lung demonstrating diffuse alveolar damage. *A*, Lung demonstrating the exudative phase, characterized by fluid accumulation in the alveolar spaces (*asterisk*) and early membrane formation (*arrow*). *B*, Lung demonstrating the organizing phase with interstitial proliferation of fibroblasts (*asterisk*).

**Figure 2.**
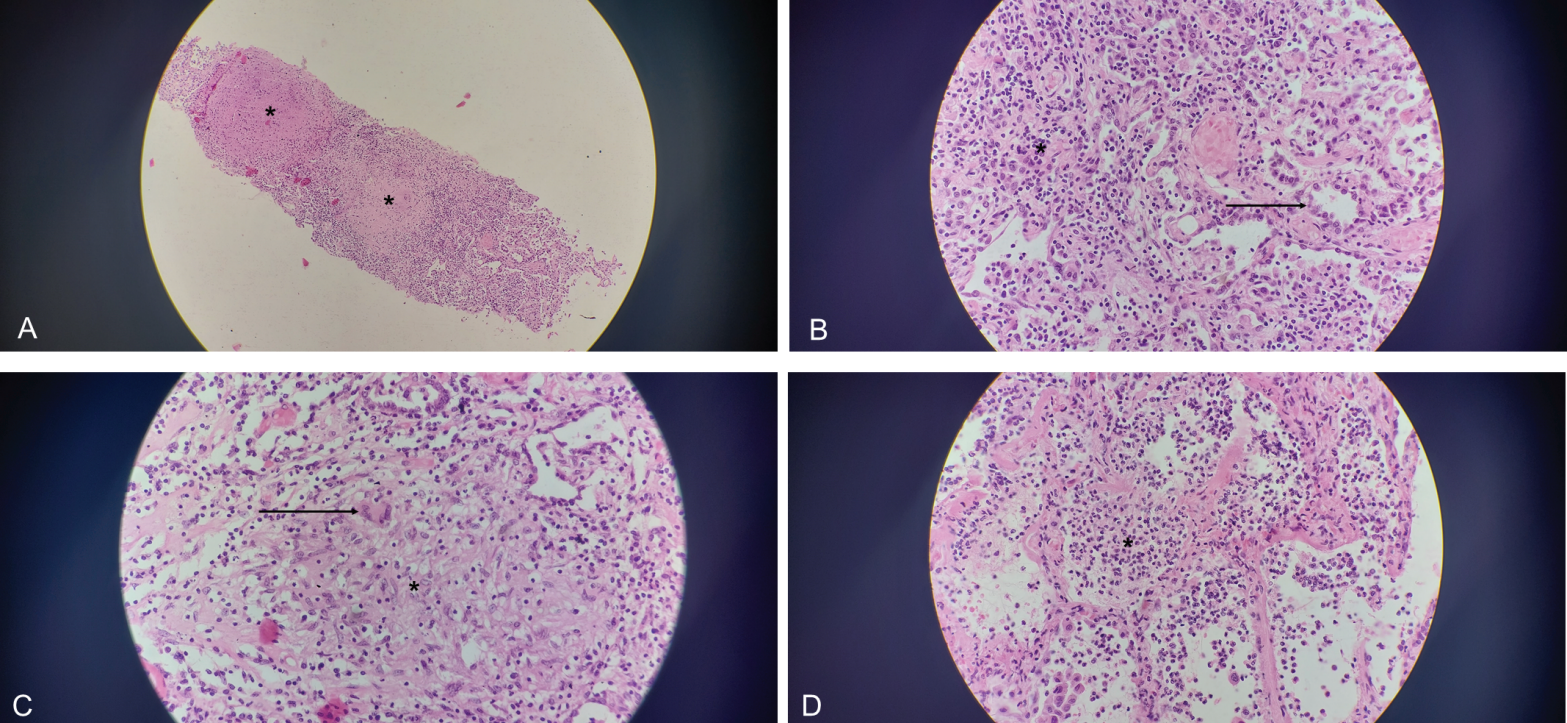
Four panels showing lung tissue with hematoxylin and eosin staining. *A*, Lung tissue at ×100 magnification. Areas of caseous necrosis (*asterisk*). *B*, Lung tissue at ×400 magnification. There is an area of pneumocyte hyperplasia (*arrow*) with lymphocytic infiltrate in the interstitium (*asterisk*). *C*, Lung tissue at ×400 magnification. There is a granuloma (*asterisk*) with a multinucleated giant cell (*arrow*). *D*, Lung tissue at ×400 magnification. There is an acute pneumonia with alveolar neutrophils (*asterisk*).

Of all the lung tissue samples collected, 7/28 were histologically normal, regardless of COVID-19 status. Among the COVID-19+ cases, 6/9 had pathological findings. Three cases (no. 2, 3, and 5) showed diffuse alveolar damage (DAD); 1 was in the acute phase and the others in the organizing phase (Figure 1). One case (no. 2) also had an acute bronchopneumonia and a caseating granulomatous inflammation with Langhans type giant cells (Figure 2), consistent with their history of pulmonary tuberculosis. By contrast, among the COVID-19− participants, only 2 cases showed DAD, 1 in the acute phase and 1 in the organizing phase. There was histological evidence of pneumonia in 6/19 COVID-19− cases and evidence of pneumonitis in 4/19 cases. Thus, it appeared that pulmonary pathology generally, and DAD more specifically, was more common among the COVID-19+ participants.

### Liver

**Figure 3.**
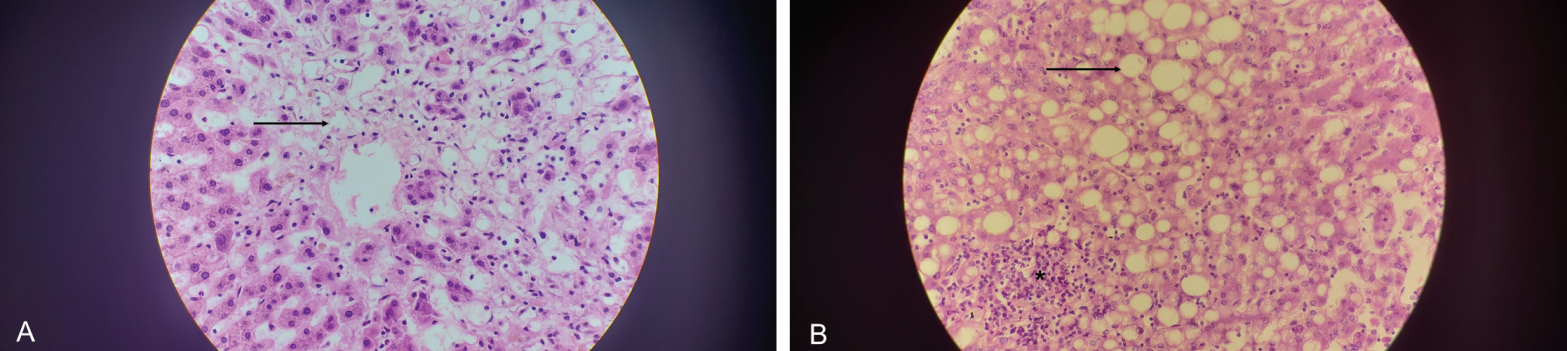
Two panels showing liver tissue with hematoxylin and eosin staining at ×100 magnification. *A*, Areas of centrilobar hepatocyte dropout, necrosis (*arrow*). *B*, Steatosis (*arrow*) and areas of acute hepatitis (*asterisk*).

Liver samples were collected from 9/9 of the COVID-19+ cases and 18/19 of the COVID-19− cases. Among the COVID-19+ cases, pathological findings were present in 8/9 cases. Steatosis was observed in 4 cases (no. 2, 3, 7, and 8). An acute hepatitis was seen in 2 cases (no. 2 and 9) (Figure 3). Other findings observed in 2 or more cases included focal interface hepatitis (no. 1 and 5) and portal tract chronic inflammation (no. 2, 3, and 9). There was 1 case of non-caseating granulomata (no. 5). In contrast, 9/19 liver tissue samples from the COVID-19- cases were histologically normal.

Among the COVID-19 negative cases, the most common finding was portal tract inflammation, which was present in 5 cases. Steatosis was observed in 2 cases. For 1 case (no. 23) the liver was not sampled. Although steatosis is a nonspecific finding present in many individuals with severe disease, the presence of 1 or more liver abnormalities appeared far more common among the COVID-19+ group (PR 1.69, 95% CI 1.04–2.74).

### Kidneys

**Figure 4.**
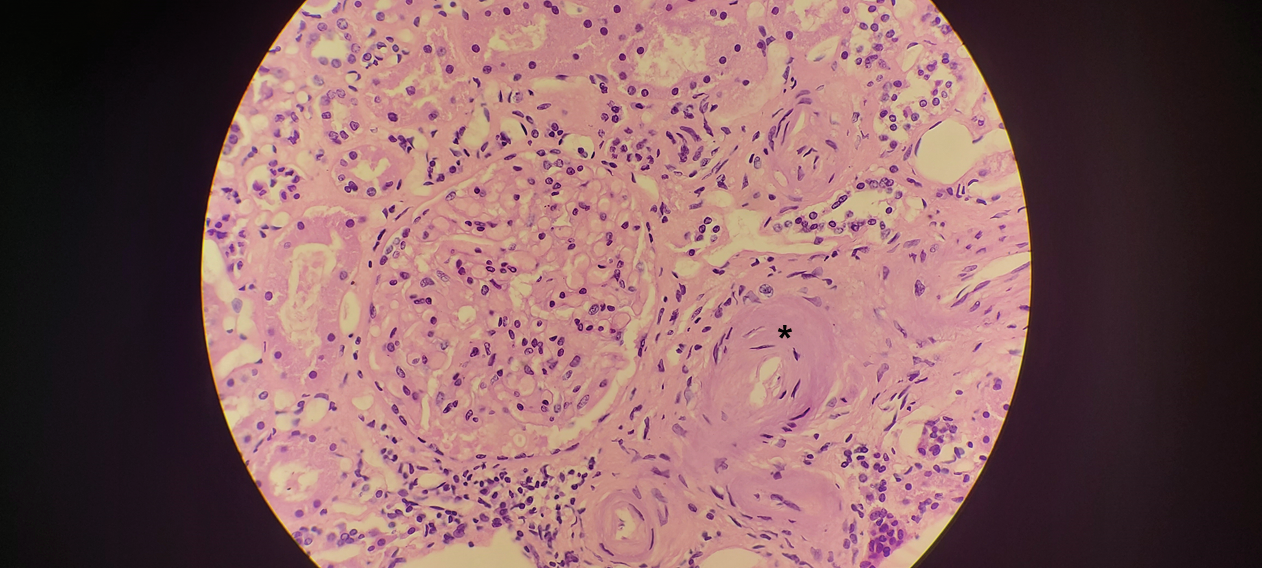
Kidney tissue with hematoxylin and eosin staining at ×100 magnification with evidence of hyaline arteriolosclerosis (*asterisk*).

We managed to collect kidney tissue from 20/28 cases, with failed sampling due to difficulty in locating the kidney based on external anatomic landmarks using the biopsy needle. We were unable to collect kidney samples from 4/19 COVID-19− cases for this reason. Among the 9 COVID-19+ cases, 4 cases could not be sampled. Among the 5 that were sampled, 4 had abnormal histological findings. The most common finding was acute kidney injury (AKI). AKI was evident in 3 cases (no. 3, 4 and 5), and all cases were focal. One case (no. 9) showed hyperplastic blood vessels (Figure 4). In contrast with the majority of kidney samples from the COVID-19+ cases, which had histological abnormalities, most kidney samples from the COVID-19− cases were normal. Among the small number of COVID-19- cases with histological findings, 1 had evidence of AKI, 1 had hyperplastic blood vessels, and 3 others showed evidence of interstitial chronic inflammation.

Overall, we detected a higher proportion of kidney abnormalities in the COVID-19+ group compared with the COVID-19− group (PR 2.4, 95% 1.04–5.56). Although these findings are nonspecific, they suggest that the kidney may be indirectly harmed during the COVID-19 syndrome, even if such injuries are not mediated directly by COVID-19 itself.

### Other Organs

**Figure 5.**
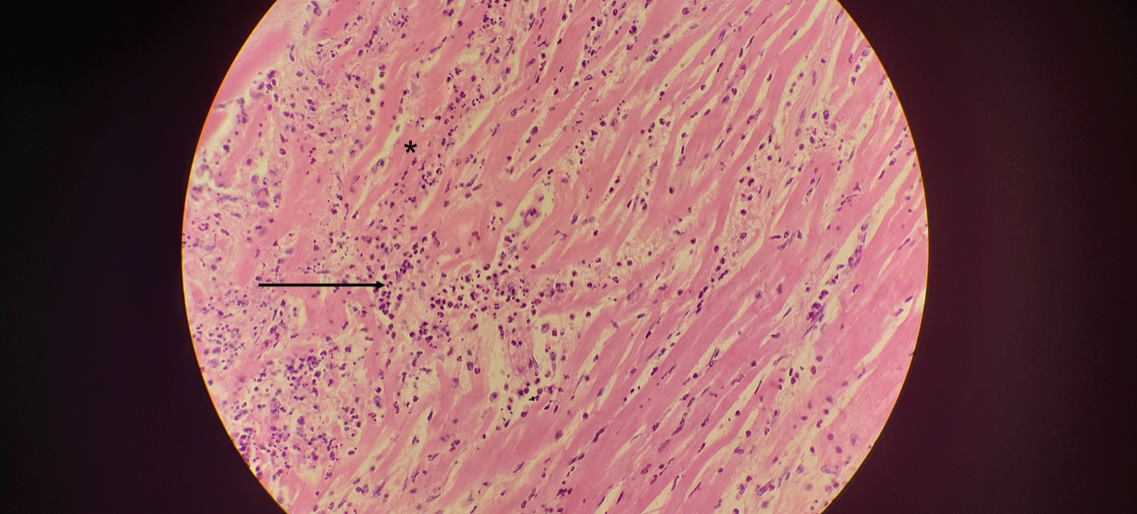
Heart tissue with hematoxylin and eosin staining at ×100 magnification showing wavy coagulative necrosis of cardiomyocytes (*asterisk*).

Because all MITS samples were taken blindly, other tissues were inadvertently sampled. Some of the tissues sampled included the heart, spleen, and pancreas. Most of these tissue samples were nondiagnostic with 1 exception. There was 1 COVID-19+ participant with a sample of heart tissue (no. 8) that showed evidence of a myocardial infarction (Figure 5).

## DISCUSSION

In this postmortem analysis of pathological specimens obtained using MITS from patients who died of a respiratory syndrome, we observed a constellation of abnormalities from lung, liver, and kidney samples that distinguished individuals with PCR-confirmed COVID-19 disease. Although our sample size was small, precluding precise estimates of differences in the distribution of abnormalities, several broad differences were observed. Among the COVID-19+ group, lung pathologies in general, and DAD more specifically, were more common than in the COVID-19− group. These findings are consistent with the clinical COVID-19 syndrome, even though the finding of DAD occurs in response to a wide range of viral pathogens and some noninfectious etiologies as well and is not unique to COVID-19 [19]. Nonetheless, it appears that diffuse alveolar damage is a common lung injury pattern that occurs as part of the COVID-19 syndrome. For 3 of our COVID-19+ with respiratory symptoms, the lung biopsies showed no evidence of pathology. This could be a limitation of the MITS sampling technique. One way to address this issue would be to increase the number of tissue biopsies harvested from the lung. Another possibility is simply that these individuals could have died from a respiratory pathogen other than SARS-CoV-2. Immunohistochemistry or in situ hybridization could also be used to provide further evidence of tissue invasion.

It is worth noting that in many of the COVID-19 negative cases, there was also evidence of pneumonia and pneumonitis. Because MITS eligibility was based on the presence of respiratory symptoms as part of the illness leading to death, it is quite possible that these individuals were infected with a respiratory pathogen other than SARS-CoV-2 at the time of their death. Another potential explanation for this observation could be the distribution of underlying risk factors amongst the COVID-19− cases. Consistent with our prior reports of COVID-19 in this population, hypertension, HIV/AIDS, and tuberculosis were present more often among the COVID-19+ than COVID-19− participants [6].

In Sub-Saharan Africa, very few COVID-19 autopsy studies have been done, despite a high number of COVID-19-related deaths. To our knowledge, there has been only 1 previous published study from an African setting, and that paper described postmortem autopsy findings in a South African cohort [16]. Our evaluation of the lung tissue samples showed histologic abnormalities in both COVID-19+ and COVID-19− cases. SARS-CoV-2 infection of the lung can present with various histological appearances depending on the stage of progression of the infection including pneumonia, interstitial inflammation with pneumocyte damage/hyperplasia, and intra alveolar exudate (with or without organization). Immunohistochemistry and/or in situ hybridization could provide further evidence that COVID-19 plays a direct role in these injuries, as opposed to setting in motion a series of pathophysiological events that indirectly result from COVID-19 disease. Histologically the pneumonia/pneumonitis in both COVID-19+ and COVID-19− cases showed no notable differences suggesting the same pathway in lung responses. Thus, there was no evident pathognomonic feature unique to this virus.

Studies have highlighted a common vascular thrombotic response in COVID-19 infections [19]. And yet only 1 COVID-19+ case in our sample demonstrated thrombi in pulmonary vessels. This may reflect a limitation of the MITS approach, because the core tissue biopsies sample only a small portion of the lung and could easily miss such infarcts if they are distributed sporadically and/or at low density. Other teams have also reported on the low rate of thrombus detection via MITS among victims of COVID-19 [20].

This study also revealed histological involvement of liver and kidney. The liver sections, regardless of COVID-19 infections status, showed steatosis and inflammation which was largely non-specific in nature. It is worth noting that 3/4 COVID-19+ cases with steatosis occurred in individuals who were living with HIV. Although antiretroviral drugs can cause steatosis, the treatment status of these individuals was unavailable to us. Because we did not observe any steatosis among individuals with HIV who were COVID-19−, we can infer the observed steatosis was likely due to COVID-19 disease. Other studies have also reported higher frequencies of steatosis among COVID-19+ individuals compared to COVID-19− controls [21].

In the kidneys there was evidence of acute tubular changes, glomerular sclerosis and vascular alterations. These changes have been described previously and are not specific to COVID-19+ cases, leading us to attribute the observed alterations to pre-existing conditions and/or to the severe illnesses that preceded death, rather than COVID-19 infection [20, 22–24].

Regardless of COVID-19 infection status, there were several cases in which there was no evidence of pathology. It is known that inflammation in the lung can be patchy in distribution, which could allow areas of pathology to be missed during the sampling process. As such, obtaining a larger number of core biopsies could be helpful, especially from the lung. In some cases, we were unable to yield a tissue sample from the target organ. This was a particular challenge when collecting kidney tissue via MITS. Ultrasound could be a valuable and complementary technology when conducting MITS by helping to guide the biopsy needle towards the organ of interest.

## LIMITATIONS

There are several limitations to our study. First, our sample size was small, which precludes firm conclusions about the distribution of underlying risk factors and pathologic findings. However, the small number of samples we did collect were of high quality and adequate for diagnosing pathology in nearly all cases. Second, symptoms and risk factors were collected from next of kin and medical chart data (facility deaths only). The accuracy of these data is subject to recall bias, nonmedical observer status, and limited by the accuracy of medical charts. Unfortunately, the solid data on these conditions, including the duration, severity, or treatment was unavailable in our study. Third, we reported high frequencies of respiratory symptoms in this cohort. Because these symptoms were used to screen cases for eligibility, these will be overrepresented in our sample by definition. Finally, we have not applied additional diagnostic techniques (PCR, immunohistochemistry, and/or electron microscopy) to identify SARS-CoV-2 virus in the tissues sampled. Our team is currently looking to incorporate immunohistochemistry in a forthcoming analysis.

## CONCLUSION

Our current work highlights the utility of MITS for understanding pathology associated with COVID-19 postmortem. In the majority of cases, the tissue samples collected from the lung were adequate for definitive histological diagnosis. This analysis strengthens our understanding of the pathology of COVID-19 and takes us one step closer to determining if COVID-19 was the cause of death or rather part of the causal pathway. Additional work needs to be done before a death can be attributed to COVID-19 with a high degree of certainty.

## Supplementary Data

Supplementary materials are available at *Clinical Infectious Diseases* online. Consisting of data provided by the authors to benefit the reader, the posted materials are not copyedited and are the sole responsibility of the authors, so questions or comments should be addressed to the corresponding author.

ciab858_suppl_Supplementary_Table_1

## Notes

***Acknowledgments.*** We would like to acknowledge the families in Zambia who suffered a great loss and were willing to participant in our studies.

***Financial sources.*** The ZPRIME study and the COVID-19 expansion were made possible through the generous support of the Bill and Melinda Gates Foundation (grant number OPP1163027). The MITS substudy was supported by the MITS Surveillance Alliance, funded by the Bill and Melinda Gates Foundation (grant number OPP1180554). Under the grant conditions of the Foundation, a Creative Commons Attribution 4.0 Generic License has already been assigned to the Author Accepted Manuscript version that might arise from this submission.

***Supplement sponsorship.*** This supplement is sponsored by MITS Surveillance Alliance Secretariat, led by RTI International, with funding from the Bill and Melinda Gates Foundation.

***Potential conflicts of interest.*** The authors: No reported conflicts of interest. All authors have submitted the ICMJE Form for Disclosure of Potential Conflicts of Interest. Conflicts that the editors consider relevant to the content of the manuscript have been disclosed.

## References

[1] Cucinotta D, Vanelli M. WHO declares COVID-19 a pandemic. Acta Biomed 2020; 91:157–60. 32191675 10.23750/abm.v91i1.9397 PMC7569573

[2] Zhu N, Zhang D, Wang W, et al. A novel coronavirus from patients with pneumonia in China, 2019. N Engl J Med 2020; 382:727–33. 31978945 10.1056/NEJMoa2001017 PMC7092803

[3] World Health Organization. WHO COVID-19 dashboard. Available at: https://covid19.who.int/.

[4] Simulundu E, Mupeta F, Chanda-Kapata P, et al. First COVID-19 case in Zambia: comparative phylogenomic analyses of SARS-CoV-2 detected in African countries. Int J Infect Dis 2021; 102:455–9. 33035675 10.1016/j.ijid.2020.09.1480 PMC7537667

[5] Zambia National Public Health Institute. Zambia COVID-19 situation report no. 161. Lusaka: National Public Health Institute, 2021.

[6] Mwananyanda L, Gill CJ, MacLeod W, et al. Covid-19 deaths in Africa: prospective systematic postmortem surveillance study. BMJ 2021; 372:n334. 33597166 10.1136/bmj.n334 PMC7887952

[7] Huang C, Wang Y, Li X, et al. Clinical features of patients infected with 2019 novel coronavirus in Wuhan, China. Lancet 2020; 395:497–506. 31986264 10.1016/S0140-6736(20)30183-5 PMC7159299

[8] Chen N, Zhou M, Dong X, et al. Epidemiological and clinical characteristics of 99 cases of 2019 novel coronavirus pneumonia in Wuhan, China: a descriptive study. Lancet 2020; 395:507–13. 32007143 10.1016/S0140-6736(20)30211-7 PMC7135076

[9] Salerno M, Sessa F, Piscopo A, et al. No Autopsies on COVID-19 deaths: a missed opportunity and the lockdown of science. J Clin Med 2020; 9:1472. 10.3390/jcm9051472 PMC7291342 32422983

[10] Hanley B, Lucas SB, Youd E, Swift B, Osborn M. Autopsy in suspected COVID-19 cases. J Clin Pathol 2020; 73:239–42. 32198191 10.1136/jclinpath-2020-206522

[11] D’Onofrio V, Donders E, Vanden Abeele ME, et al. The clinical value of minimal invasive autopsy in COVID-19 patients. PLoS One 2020; 15:e0242300. 33175911 10.1371/journal.pone.0242300 PMC7657516

[12] Maixenchs M, Anselmo R, Sanz A, et al. Healthcare providers’ views and perceptions on post-mortem procedures for cause of death determination in Southern Mozambique. PLoS One 2018; 13:e0200058. 29979720 10.1371/journal.pone.0200058 PMC6034841

[13] Castillo P, Martínez MJ, Ussene E, et al. Validity of a minimally invasive autopsy for cause of death determination in adults in Mozambique: an observational study. PLoS Med 2016; 13:e1002171. 27875530 10.1371/journal.pmed.1002171 PMC5119723

[14] Martínez MJ, Massora S, Mandomando I, et al. Infectious cause of death determination using minimally invasive autopsies in developing countries. Diagn Microbiol Infect Dis 2016; 84:80–6. 26508103 10.1016/j.diagmicrobio.2015.10.002

[15] Paganelli CR, Goco NJ, McClure EM, et al. The evolution of minimally invasive tissue sampling in postmortem examination: a narrative review. Glob Health Action 2020; 13:1792682. 32713325 10.1080/16549716.2020.1792682 PMC7480574

[16] Bruce-Brand C, Allwood BW, Koegelenberg CFN, et al. Postmortem lung biopsies from four patients with COVID-19 at a tertiary hospital in Cape Town, South Africa. S Afr Med J 2020; 110:1195–200. 33403965 10.7196/SAMJ.2020.v110i12.15290

[17] Esona MD, McDonald S, Kamili S, Kerin T, Gautam R, Bowen MD. Comparative evaluation of commercially available manual and automated nucleic acid extraction methods for rotavirus RNA detection in stools. J Virol Methods 2013; 194:242–9. 24036075 10.1016/j.jviromet.2013.08.023 PMC4603280

[18] Jeddi F, Piarroux R, Mary C. Application of the NucliSENS easyMAG system for nucleic acid extraction: optimization of DNA extraction for molecular diagnosis of parasitic and fungal diseases. Parasite 2013; 20:52. 24331004 10.1051/parasite/2013051 PMC3859032

[19] Deshmukh V, Motwani R, Kumar A, Kumari C, Raza K. Histopathological observations in COVID-19: a systematic review. J Clin Pathol 2021; 74:76–83. 32817204 10.1136/jclinpath-2020-206995

[20] Vasquez-Bonilla WO, Orozco R, Argueta V, et al. A review of the main histopathological findings in coronavirus disease 2019. Hum Pathol 2020; 105:74–83. 32750378 10.1016/j.humpath.2020.07.023 PMC7395947

[21] Medeiros AK, Barbisan CC, Cruz IR, et al. Higher frequency of hepatic steatosis at CT among COVID-19-positive patients. Abdom Radiol (NY) 2020; 45:2748–54. 32683613 10.1007/s00261-020-02648-7 PMC7368629

[22] Díaz LA, Idalsoaga F, Cannistra M, et al. High prevalence of hepatic steatosis and vascular thrombosis in COVID-19: a systematic review and meta-analysis of autopsy data. World J Gastroenterol 2020; 26:7693–706. 33505145 10.3748/wjg.v26.i48.7693 PMC7789052

[23] Schmit G, Lelotte J, Vanhaebost J, Horsmans Y, Van Bockstal M, Baldin P. The liver in COVID-19-related death: protagonist or innocent bystander? Pathobiology 2021; 88:88–94. 33108789 10.1159/000512008 PMC7705929

[24] Rudnick MR, Hilburg R. Acute kidney injury in COVID-19: another challenge for nephrology. Am J Nephrol 2020; 51:761–3. 33059350 10.1159/000511161 PMC7649678

