# Supplementary material for: Histopathological Evaluation of Deceased Persons in Lusaka, Zambia With or Without Coronavirus Disease 2019 (COVID-19) Infection: Results Obtained From Minimally Invasive Tissue Sampling
Source: Clin Infect Dis. 2021 Dec 15;73(Suppl 5):S465–71. doi: 10.1093/cid/ciab858 (PMC8672753; doi:10.1093/cid/ciab858)
Supplement: ciab858_suppl_Supplementary_Table_1 [file ciab858_suppl_supplementary_table_1.docx]

**Supplementary Table 1.** Summary of key histologic findings among CV19 positive and negative individuals by organ

4A. Histological findings of **lung tissue** biopsies

| **Lung** | | | | | | | | | | | |
| --- | --- | --- | --- | --- | --- | --- | --- | --- | --- | --- | --- |
| **Case** | **Normal** | **DAD Acute Phase** | **DAD**  **Organizing Phase** | **DAD**  **Fibrotic Phase** | **Viral**  **cytopathic changes** | **Pneumonia** | **Pneumonitis chronic interstitial inflammation** | **Thrombi** | **Caseating granulomata** | **Engorged vessels** | **Macrophages in alveoli** |
| COVID-19 positive | | | | | | | | | | | |
| 1 |  |  |  |  |  |  | X (mild) |  |  |  |  |
| 2 |  | X |  |  |  | X (acute) |  | X | X |  |  |
| 3 |  |  | X |  |  |  |  |  |  |  |  |
| 4 | X |  |  |  |  |  |  |  |  |  |  |
| 5 |  |  | X |  |  |  |  |  |  |  |  |
| 6 | X |  |  |  |  |  |  |  |  |  |  |
| 7 | X |  |  |  |  |  |  |  |  |  |  |
| 8 |  |  |  |  |  |  |  |  |  | X | X |
| 9 |  |  |  |  |  |  |  |  |  | X |  |
| COVID-19 negative | | | | | | | | | | | |
| 10 |  | X |  |  |  | X |  |  |  |  |  |
| 11 | X |  |  |  |  |  |  |  |  |  |  |
| 12 |  |  |  |  |  |  |  |  | X |  |  |
| 13 |  |  |  |  |  | X |  |  |  |  |  |
| 14 |  |  |  |  |  |  |  |  |  | X |  |
| 15 |  |  |  |  |  |  |  |  |  | X |  |
| 16 | X |  |  |  |  |  |  |  |  |  |  |
| 17 |  |  |  |  |  | X |  |  |  |  |  |
| 18 |  |  |  |  |  | X |  |  |  |  |  |
| 19 | X |  |  |  |  |  |  |  |  |  |  |
| 20 |  |  |  |  |  | X |  |  | X |  |  |
| 21 | X |  |  |  |  |  |  |  |  |  |  |
| 22 |  |  |  |  |  |  | X |  |  |  |  |
| 23 |  |  |  |  |  |  | X |  |  |  |  |
| 24 |  |  |  |  |  |  | X |  |  |  | X |
| 25 |  |  |  |  |  | X |  |  |  |  |  |
| 26 |  |  |  |  |  |  | X |  |  |  | X |
| 27 |  |  | X |  |  |  |  |  |  |  | X |
| 28 |  |  |  |  |  |  |  | X |  | X |  |

Abbreviation: DAD, diffuse alveolar damage.

4B. Histological findings of **liver tissue** biopsies

| **Liver** | | | | | | | | | | |
| --- | --- | --- | --- | --- | --- | --- | --- | --- | --- | --- |
| **Case** | **Not**  **sampled** | **Normal** | **Steatosis** | **Chronic lymphatic inflammation** | **Congestion** | **Focal interface hepatitis** | **Portal tract**  **inflammation (lymphocytic)** | **Non caseating granulomas** | **Necrosis (patchy)** | **Acute**  **hepatitis** |
| COVID-19 positive | | | | | | | | | | |
| 1 |  |  |  |  |  | X  (focal/mild) |  |  |  |  |
| 2 |  |  | X |  |  |  | X |  |  | X |
| 3 |  |  | X |  |  |  | X | X |  |  |
| 4 |  |  |  |  |  |  |  |  |  |  |
| 5 |  |  |  |  |  | X (foci) |  |  | X |  |
| 6 |  | X |  |  |  |  |  |  |  |  |
| 7 |  |  | X | X (focal) | X |  |  |  |  |  |
| 8 |  |  | X |  |  |  |  |  |  |  |
| 9 |  |  |  |  |  |  | X |  |  | X |
| COVID-19 negative | | | | | | | | | | |
| 10 |  | X |  |  |  |  |  |  |  |  |
| 11 |  | X |  |  | X (mild) |  |  |  |  |  |
| 12 |  |  |  |  |  |  |  |  | X |  |
| 13 |  |  |  |  |  |  | X |  |  |  |
| 14 |  |  |  |  |  |  |  |  |  | X (mild) |
| 15 |  | X |  |  |  |  |  |  |  |  |
| 16 |  | X |  |  |  |  |  |  |  |  |
| 17 |  | X |  |  |  |  |  |  |  |  |
| 18 |  |  | X |  |  |  |  |  |  |  |
| 19 |  | X |  |  |  |  |  |  |  |  |
| 20 |  |  |  |  |  |  | X |  |  |  |
| 21 |  |  |  |  | X |  |  |  |  |  |
| 22 |  |  | X |  |  |  | X |  |  |  |
| 23 | X |  |  |  |  |  |  |  |  |  |
| 24 |  | X |  |  |  |  |  |  |  |  |
| 25 |  |  |  |  |  |  | X |  |  |  |
| 26 |  | X |  |  |  |  |  |  |  |  |
| 27 |  |  |  |  |  |  | X |  |  |  |
| 28 |  | X |  |  |  |  |  |  |  |  |

4C. Histological findings of **kidney tissue** biopsies

| **Case** | **Not**  **sampled** | **Normal** | **Periglomerular**  **fibrosis** | **Acute Kidney Injury (ATN/ATI)** | **Hyperplastic blood vessels** | **Glomerular collapse** | **Thrombi** | **Kidney**  **interstitial chronic inflammation** |
| --- | --- | --- | --- | --- | --- | --- | --- | --- |
| COVID-19 positive | | | | | | | | |
| 1 | X |  |  |  |  |  |  |  |
| 2 |  | X |  |  |  |  |  |  |
| 3 |  |  |  | X (focal) |  |  |  |  |
| 4 |  |  |  | X (focal) |  |  |  |  |
| 5 |  |  |  | X (mild/focal) |  |  |  |  |
| 6 | X |  |  |  |  |  |  |  |
| 7 | X |  |  |  |  |  |  |  |
| 8 | X |  |  |  |  |  |  |  |
| 9 |  |  |  |  | X |  |  |  |
| COVID-19 negative | | | | | | | | |
| 10 | X |  |  |  |  |  |  |  |
| 11 | X |  |  |  |  |  |  |  |
| 12 | X |  |  |  |  |  |  |  |
| 13 |  | X |  |  |  |  |  |  |
| 14 |  |  |  | X |  |  |  |  |
| 15 |  | X |  |  |  |  |  |  |
| 16 |  | X |  |  |  |  |  |  |
| 17 |  | X |  |  |  |  |  |  |
| 18 | X |  |  |  |  |  |  |  |
| 19 |  |  |  |  |  |  |  | X |
| 20 |  |  |  |  |  |  |  | X |
| 21 |  |  |  |  | X |  |  |  |
| 22 |  | X |  |  |  |  |  |  |
| 23 |  |  |  |  |  |  |  | X |
| 24 |  | X |  |  |  |  |  |  |
| 25 |  | X |  |  |  |  |  |  |
| 26 |  | X |  |  |  |  |  |  |
| 27 |  | X |  |  |  |  |  |  |
| 28 |  | X |  |  |  |  |  |  |

Abbreviations: ATI, acute tubular injury; ATN, acute tubular necrosis.
